# *rac*-3-(4-Hy­droxy­benz­yl)chroman-4-one

**DOI:** 10.1107/S1600536813014645

**Published:** 2013-06-08

**Authors:** S. Shalini, C. R. Girija, Lalitha Simon, K. K. Srinivasan, T. V. Venkatesha

**Affiliations:** a Department of Chemistry, Chemistry Research Centre (Affiliated to Kuvempu University), SSMRV Degree College, Jayanagar 4th T Block, Bangalore 560 041, India; b Department of Chemistry, KMC International Centre, Manipal University, Manipal 576 104, India; c Department of Pharmaceutical Chemistry, Manipal College of Pharmaceutical Sciences, Manipal University, Manipal 576 104, India; d Department of Chemistry, Jnana Sahyadri, Kuvempu University, Shankargatta 577 451, India

## Abstract

In the racemic title compound, C_16_H_14_O_3_, the ring of the 4-hy­droxy­benzyl substituent group forms a dihedral angle of 80.12 (12)° with the benzene ring of the chromanone system. Two C atoms of the pyran­one ring and the H atoms on the benzyl α-C atom are disordered over two sites, with site-occupation factors of 0.818 (8) and 0.182 (8). The crystal structure is stabilized by O—H⋯O hydrogen bonds, which form parallel one-dimensional zigzag chains down the *c* axis and are inter­connected by both methine C—H⋯O hydrogen bonds and weak aromatic C—H⋯π inter­actions, giving a sheet structure lying parallel to [011].

## Related literature

For general background on the properties of isoflavanones (derivatives of 3-benzyl-4*H*-chromen-4-one), see: Klymchenko *et al.* (2003 ▶); Sengupta & Kasha (1979 ▶). For related structures, see: Etter *et al.* (1986 ▶); Waller *et al.* (2003 ▶); Wera *et al.* (2011 ▶); Shalini *et al.* (2013 ▶). For inter­molecular inter­actions, see: Takahashi *et al.* (2001 ▶). For ring-puckering calculations, see: Cremer & Pople (1975 ▶).
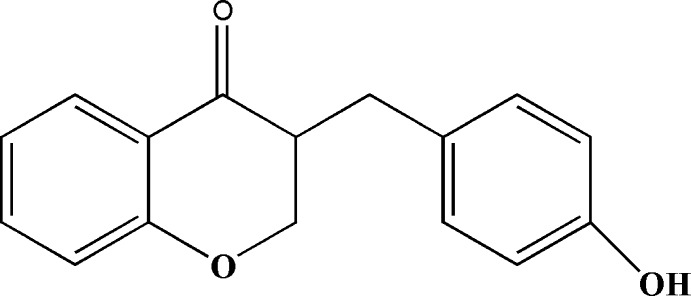


## Experimental

### 

#### Crystal data

C_16_H_14_O_3_
*M* *_r_* = 254.27
Monoclinic, 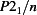

*a* = 5.2570 (2) Å
*b* = 17.0254 (7) Å
*c* = 14.6879 (5) Å
β = 97.806 (2)°
*V* = 1302.42 (9) Å^3^
*Z* = 4
Mo *K*α radiation
μ = 0.09 mm^−1^
*T* = 293 K
0.30 × 0.20 × 0.20 mm

#### Data collection

Bruker Kappa APEX2 CCD diffractometer
Absorption correction: multi-scan (*SADABS*; Bruker, 2004 ▶) *T*_min_ = 0.962, *T*_max_ = 0.991
12297 measured reflections
2288 independent reflections
1523 reflections with *I* > 2σ(*I*)
*R*_int_ = 0.026

#### Refinement

*R*[*F* ^2^ > 2σ(*F* ^2^)] = 0.041
*wR*(*F* ^2^) = 0.110
*S* = 1.11
2288 reflections
187 parameters
5 restraints
H-atom parameters constrained
Δρ_max_ = 0.13 e Å^−3^
Δρ_min_ = −0.11 e Å^−3^

### 

Data collection: *APEX2* (Bruker, 2004 ▶); cell refinement: *APEX2* and *SAINT* (Bruker, 2004 ▶); data reduction: *SAINT* and *XPREP* (Bruker, 2004 ▶); program(s) used to solve structure: *SIR92* (Altomare *et al.*, 1993 ▶); program(s) used to refine structure: *SHELXL97* (Sheldrick, 2008 ▶); molecular graphics: *ORTEP-3 for Windows* (Farrugia, 2012 ▶); software used to prepare material for publication: *SHELXL97*.

## Supplementary Material

Crystal structure: contains datablock(s) I, global. DOI: 10.1107/S1600536813014645/zs2259sup1.cif

Structure factors: contains datablock(s) I. DOI: 10.1107/S1600536813014645/zs2259Isup2.hkl

Supplementary material file. DOI: 10.1107/S1600536813014645/zs2259Isup3.cml

Additional supplementary materials:  crystallographic information; 3D view; checkCIF report

## Figures and Tables

**Table 1:** Hydrogen-bond geometry (Å, °) *Cg*1 is the centroid of the C1–C6 ring.

*D*—H⋯*A*	*D*—H	H⋯*A*	*D*⋯*A*	*D*—H⋯*A*
O1—H1⋯O2^i^	0.82	1.94	2.752 (2)	173
C8—H8⋯O1^ii^	0.98	2.39	3.166 (4)	136
C16—H16⋯*Cg*1^iii^	0.93	3.14	4.022 (3)	159

Symmetry codes: (i) 
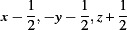
; (ii) 
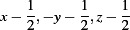
; (iii) 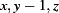.

**Fig. 1.**
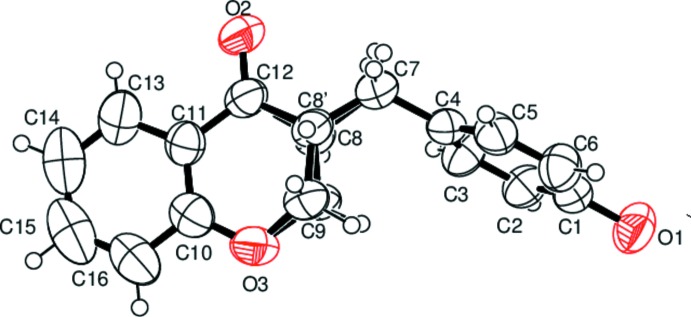
The molecular structure of the title compound, showing the atom-labelling scheme. Displacement ellipsoids are drawn at the 40% probability level.

**Fig. 2.**
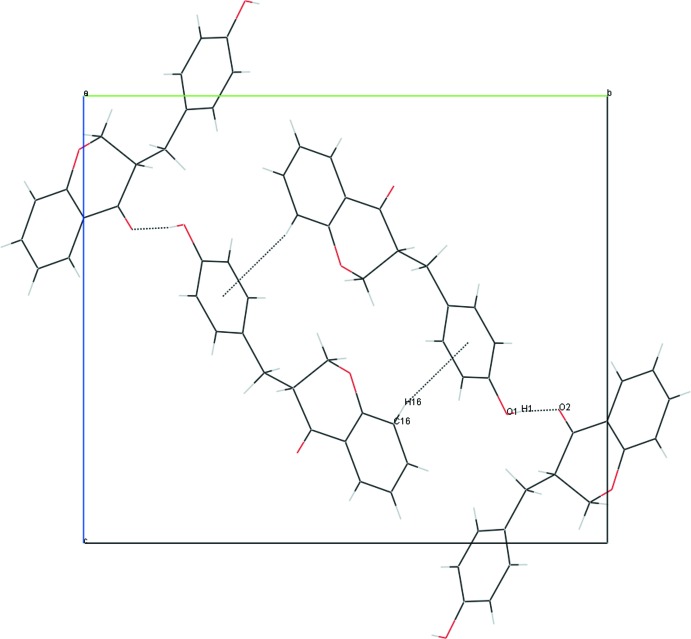
The intermolecular interactions, viewed down the a cell direction. The O—H···O and C—H···π hydrogen bonds are represented by dashed lines and the minor disordered atoms C8' and C9' are not shown.

## supplementary crystallographic information

### Comment

Naturally occurring homoisoflavanones that possess a 3-benzyl-substituted chroman ring system as a common framework have been isolated from a wide range of natural sources and exhibit a variety of biological activities (Wera *et al.* (2011). Here we report the structure of the racemic chroman-4-one benzyl derivative, C_16_H_14_O_3_, in which the phenyl ring of the 4-hydroxybenzyl substituent group forms a dihedral angle of 80.12 (12)° with the phenyl ring of the chromanone ring (Fig. 1). The ring-puckering parameters q_2_ = 0.3745 (0) Å, q_3_ = -0.2819 (0) Å, Q_T_ = 0.4688 Å and φ = -144.45 (0)° of the 2,3-dihydro-4*H*-chroman-4-one ring are indicative of an envelope conformation. Two carbon atoms of the pyranone ring (C8 and C9) with the hydrogen atoms of the benzyl C-atom (C7) are disordered over two sites with site-occupation factors of 0.818 (8) (C8, C9) and 0.182 (8) (C8' C9').

In the crystal a strong intermolecular hydrogen bond (O1—H1···O2^i^) (Table 1) results in the formation of one-dimensional zigzag chains which extend along *c* (Fig. 2). Weak intermolecular methine C8—H···O1^ii^ hydrogen bonds and weak aromatic C16—H···π (C1–C6)^iii^ interactions [H···*Cg* = 3.139 Å] [symmetry code (iii) *x*, *y* - 1, *z*] give sheets extending along [011]. There are no π–π stacking interactions present in the structure.

### Experimental

In the preparation of the title compound, 2'-hydroxydihydrochalcone (0.1 g, 1 equivalent) was dissolved in ethanol (10 ml) was refluxed with paraformaldehyde (0.022 g, 2 equivalents) and 50% aqueous diethylamine (0.2 ml, 1 equivalent) for 7 h. Ethanol was distilled off and the residue was taken up in ethyl acetate. The ethyl acetate layer was washed with water and then with dilute HCL. Ethyl acetate was distilled off and the oily residue was column-chromatographed over silica using petroleum ether:ethyl acetate (7:3) as eluent to obtain the title compound in 60–70% yield. Colourless single crystals were grown in ethanol by slow evaporation at ambient temperature. Spectroscopy: IR (cm^-1^): 3282 (O—H str), 1672 (C═O str), 1604 (C═C str); mass (*m*/*z*): *M*^+^ 254 (70%), 237, 147 (100%), 121; ^1^H NMR (400 MHz,solvent DMSO): δ 4.3 (dd, J = 11.6, 4.4 Hz, 1H, 2-H), δ 4.1 (dd, J = 16, 8.8 Hz, 1H, 2-H), δ 2.5 (m, 1H, 3-H), δ 3 (m, 2H, 9-H), δ 7.7 (dd, J = 7.6, 1.2 Hz, 1H), δ 7.5 (m, 1H), δ 7 (m, 3H, Ar—H), δ 6.6 (d, J = 8.4 Hz, 2H, Ar—H)

### Refinement

Carbon-bound H atoms were positioned geometrically, with C—H = 0.93 Å (aromatic), 0.98 Å (methine) and 0.97 Å (methylene), and were constrained to ride on their parent atoms with *U*_iso_(H) = 1.2*U*_eq_(C). The phenolic H atom was located in a difference Fourier map and was also allowed to ride with *U*_iso_(H) = 1.2*U*_eq_(O). In the refinement, positional, site-occupation factors and *U*_ij_ parameters of the disordered C atoms [C8, C9 = 0.818 (8) and C8', C9' = 0.112 (8)] were refined freely. However, the EADP instruction (Sheldrick, 2008) was used to constrain the anisotropic displacement parameters (ADPs) of the disordered C atoms of the minor components to the same values as the corresponding C atoms in the principal component. Also, SUMP and DFIX restraints were used to stabilize the refinement of the disordered atoms. The occupancies of the disordered components were fixed during the final cycles of the refinement.

### Crystal data

**Table:**

C_16_H_14_O_3_	*F*(000) = 536
*M**_r_* = 254.27	*D*_x_ = 1.297 Mg m^−^^3^
Monoclinic, *P*2_1_/*n*	Mo *K*α radiation, λ = 0.71073 Å
Hall symbol: -P 2yn	Cell parameters from 3775 reflections
*a* = 5.2570 (2) Å	θ = 2.5–23.5°
*b* = 17.0254 (7) Å	µ = 0.09 mm^−^^1^
*c* = 14.6879 (5) Å	*T* = 293 K
β = 97.806 (2)°	Block, colourless
*V* = 1302.42 (9) Å^3^	0.30 × 0.20 × 0.20 mm
*Z* = 4	

### Data collection

**Table:**

Bruker Kappa APEX2 CCD diffractometer	2288 independent reflections
Radiation source: fine-focus sealed tube	1523 reflections with *I* > 2σ(*I*)
Graphite monochromator	*R*_int_ = 0.026
ω and φ scan	θ_max_ = 25.0°, θ_min_ = 2.4°
Absorption correction: multi-scan (*SADABS*; Bruker, 2004)	*h* = −6→6
*T*_min_ = 0.962, *T*_max_ = 0.991	*k* = −17→20
12297 measured reflections	*l* = −17→17

### Refinement

**Table:**

Refinement on *F*^2^	Secondary atom site location: difference Fourier map
Least-squares matrix: full	Hydrogen site location: inferred from neighbouring sites
*R*[*F*^2^ > 2σ(*F*^2^)] = 0.041	H-atom parameters constrained
*wR*(*F*^2^) = 0.110	*w* = 1/[σ^2^(*F*_o_^2^) + (0.0283*P*)^2^ + 0.4082*P*] where *P* = (*F*_o_^2^ + 2*F*_c_^2^)/3
*S* = 1.11	(Δ/σ)_max_ = 0.001
2288 reflections	Δρ_max_ = 0.13 e Å^−^^3^
187 parameters	Δρ_min_ = −0.11 e Å^−^^3^
5 restraints	Extinction correction: *SHELXL97* (Sheldrick, 2008), Fc^*^=kFc[1+0.001xFc^2^λ^3^/sin(2θ)]^-1/4^
Primary atom site location: structure-invariant direct methods	Extinction coefficient: 0.0066 (15)

### Special details

**Table:**

Geometry. All e.s.d.'s (except the e.s.d. in the dihedral angle between two l.s. planes) are estimated using the full covariance matrix. The cell e.s.d.'s are taken into account individually in the estimation of e.s.d.'s in distances, angles and torsion angles; correlations between e.s.d.'s in cell parameters are only used when they are defined by crystal symmetry. An approximate (isotropic) treatment of cell e.s.d.'s is used for estimating e.s.d.'s involving l.s. planes.
Refinement. Refinement of *F*^2^ against ALL reflections. The weighted *R*-factor *wR* and goodness of fit *S* are based on *F*^2^, conventional *R*-factors *R* are based on *F*, with *F* set to zero for negative *F*^2^. The threshold expression of *F*^2^ > σ(*F*^2^) is used only for calculating *R*-factors(gt) *etc*. and is not relevant to the choice of reflections for refinement. *R*-factors based on *F*^2^ are statistically about twice as large as those based on *F*, and *R*-factors based on ALL data will be even larger.

### Fractional atomic coordinates and isotropic or equivalent isotropic displacement parameters (Å^2^)

**Table:**

	*x*	*y*	*z*	*U*_iso_*/*U*_eq_	Occ. (<1)
C1	1.4022 (4)	−0.27353 (12)	1.13065 (15)	0.0628 (6)	
C2	1.2332 (4)	−0.28490 (13)	1.05275 (15)	0.0717 (6)	
H2	1.0932	−0.3182	1.0534	0.086*	
C3	1.2717 (5)	−0.24661 (14)	0.97306 (15)	0.0768 (7)	
H3	1.1565	−0.2551	0.9201	0.092*	
C4	1.4740 (4)	−0.19636 (12)	0.96916 (14)	0.0620 (6)	
C5	1.6411 (4)	−0.18607 (13)	1.04841 (16)	0.0685 (6)	
H5	1.7807	−0.1526	1.0481	0.082*	
C6	1.6067 (4)	−0.22434 (14)	1.12857 (16)	0.0741 (6)	
H6	1.7230	−0.2166	1.1814	0.089*	
C7	1.5106 (4)	−0.15507 (15)	0.88088 (15)	0.0770 (7)	
H7A	1.6694	−0.1253	0.8906	0.092*	0.818 (8)
H7B	1.5272	−0.1942	0.8340	0.092*	0.818 (8)
H7C	1.6938	−0.1473	0.8820	0.092*	0.182 (8)
H7D	1.4570	−0.1915	0.8312	0.092*	0.182 (8)
C8	1.2935 (8)	−0.10032 (19)	0.8465 (2)	0.0608 (9)	0.818 (8)
H8	1.1365	−0.1320	0.8389	0.073*	0.818 (8)
C9	1.2480 (9)	−0.0336 (3)	0.9090 (2)	0.0726 (11)	0.818 (8)
H9A	1.2381	−0.0543	0.9700	0.087*	0.818 (8)
H9B	1.3937	0.0018	0.9135	0.087*	0.818 (8)
C8'	1.387 (3)	−0.0801 (8)	0.8553 (13)	0.0608 (9)	0.182 (8)
H8'	1.5045	−0.0383	0.8803	0.073*	0.182 (8)
C9'	1.146 (3)	−0.0730 (10)	0.8989 (10)	0.058 (4)	0.182 (8)
H9'1	1.1852	−0.0811	0.9646	0.070*	0.182 (8)
H9'2	1.0246	−0.1130	0.8742	0.070*	0.182 (8)
C10	1.0010 (4)	0.03237 (13)	0.79097 (18)	0.0718 (6)	
C11	1.1423 (4)	−0.00044 (12)	0.72734 (15)	0.0671 (6)	
C12	1.3175 (4)	−0.06544 (13)	0.75340 (15)	0.0662 (6)	
C13	1.1050 (6)	0.02833 (16)	0.63788 (18)	0.0956 (8)	
H13	1.1988	0.0074	0.5943	0.115*	
C14	0.9319 (7)	0.0870 (2)	0.6134 (3)	0.1193 (11)	
H14	0.9071	0.1057	0.5534	0.143*	
C15	0.7942 (7)	0.11842 (19)	0.6781 (3)	0.1237 (12)	
H15	0.6769	0.1584	0.6612	0.148*	
C16	0.8271 (5)	0.09181 (16)	0.7663 (2)	0.0994 (9)	
H16	0.7333	0.1135	0.8094	0.119*	
O1	1.3727 (3)	−0.30821 (10)	1.21218 (10)	0.0888 (6)	
H1	1.2435	−0.3357	1.2055	0.107*	
O2	1.4570 (3)	−0.09234 (10)	0.70136 (10)	0.0866 (5)	
O3	1.0259 (3)	0.00860 (9)	0.88004 (12)	0.0819 (5)	

### Atomic displacement parameters (Å^2^)

**Table:**

	*U*^11^	*U*^22^	*U*^33^	*U*^12^	*U*^13^	*U*^23^
C1	0.0626 (13)	0.0639 (14)	0.0646 (13)	0.0046 (11)	0.0181 (11)	0.0075 (11)
C2	0.0703 (14)	0.0750 (15)	0.0719 (15)	−0.0148 (11)	0.0176 (12)	0.0028 (12)
C3	0.0750 (15)	0.0922 (17)	0.0625 (14)	−0.0139 (13)	0.0072 (11)	0.0019 (12)
C4	0.0578 (12)	0.0648 (14)	0.0667 (14)	0.0074 (10)	0.0207 (11)	0.0064 (11)
C5	0.0550 (12)	0.0682 (15)	0.0841 (16)	−0.0017 (10)	0.0156 (11)	0.0113 (12)
C6	0.0676 (14)	0.0841 (17)	0.0685 (14)	−0.0034 (12)	0.0015 (11)	0.0076 (12)
C7	0.0683 (14)	0.0928 (17)	0.0747 (15)	0.0110 (13)	0.0278 (12)	0.0168 (13)
C8	0.074 (2)	0.0546 (17)	0.0585 (15)	−0.0042 (14)	0.0242 (17)	−0.0011 (14)
C9	0.079 (3)	0.080 (3)	0.0608 (18)	0.007 (2)	0.0166 (17)	−0.0059 (18)
C8'	0.074 (2)	0.0546 (17)	0.0585 (15)	−0.0042 (14)	0.0242 (17)	−0.0011 (14)
C9'	0.061 (8)	0.054 (10)	0.065 (8)	−0.009 (6)	0.022 (7)	−0.008 (7)
C10	0.0660 (14)	0.0589 (14)	0.0909 (18)	−0.0062 (11)	0.0119 (13)	−0.0013 (13)
C11	0.0734 (14)	0.0571 (13)	0.0708 (15)	−0.0064 (11)	0.0097 (11)	0.0041 (11)
C12	0.0796 (15)	0.0618 (14)	0.0610 (13)	−0.0042 (11)	0.0229 (12)	−0.0044 (11)
C13	0.118 (2)	0.0855 (19)	0.0816 (18)	−0.0047 (17)	0.0078 (15)	0.0140 (15)
C14	0.134 (3)	0.096 (2)	0.119 (3)	−0.002 (2)	−0.017 (2)	0.036 (2)
C15	0.108 (3)	0.080 (2)	0.173 (4)	0.0091 (18)	−0.016 (3)	0.024 (2)
C16	0.0839 (19)	0.0734 (18)	0.140 (3)	0.0107 (15)	0.0117 (18)	0.0041 (18)
O1	0.0925 (12)	0.1042 (13)	0.0711 (10)	−0.0090 (9)	0.0164 (9)	0.0248 (9)
O2	0.1092 (13)	0.0882 (12)	0.0706 (10)	0.0128 (10)	0.0413 (9)	0.0031 (9)
O3	0.0861 (12)	0.0782 (11)	0.0868 (12)	0.0134 (9)	0.0308 (9)	−0.0065 (9)

### Geometric parameters (Å, º)

**Table:**

C1—O1	1.363 (2)	C9—H9A	0.9700
C1—C2	1.364 (3)	C9—H9B	0.9700
C1—C6	1.366 (3)	C8'—C9'	1.501 (10)
C2—C3	1.379 (3)	C8'—C12	1.513 (18)
C2—H2	0.9300	C8'—H8'	0.9800
C3—C4	1.372 (3)	C9'—O3	1.535 (15)
C3—H3	0.9300	C9'—H9'1	0.9700
C4—C5	1.371 (3)	C9'—H9'2	0.9700
C4—C7	1.510 (3)	C10—O3	1.359 (3)
C5—C6	1.379 (3)	C10—C16	1.379 (3)
C5—H5	0.9300	C10—C11	1.388 (3)
C6—H6	0.9300	C11—C13	1.391 (3)
C7—C8'	1.457 (9)	C11—C12	1.457 (3)
C7—C8	1.507 (4)	C12—O2	1.218 (2)
C7—H7A	0.9700	C13—C14	1.366 (4)
C7—H7B	0.9700	C13—H13	0.9300
C7—H7C	0.9700	C14—C15	1.378 (4)
C7—H7D	0.9700	C14—H14	0.9300
C8—C9	1.500 (4)	C15—C16	1.361 (4)
C8—C12	1.512 (4)	C15—H15	0.9300
C8—H8	0.9800	C16—H16	0.9300
C9—O3	1.388 (4)	O1—H1	0.8200
			
O1—C1—C2	122.4 (2)	O3—C9—H9A	108.8
O1—C1—C6	118.0 (2)	C8—C9—H9A	108.8
C2—C1—C6	119.6 (2)	O3—C9—H9B	108.8
C1—C2—C3	119.4 (2)	C8—C9—H9B	108.8
C1—C2—H2	120.3	H9A—C9—H9B	107.7
C3—C2—H2	120.3	C7—C8'—C9'	109.5 (10)
C4—C3—C2	122.3 (2)	C7—C8'—C12	116.1 (11)
C4—C3—H3	118.8	C9'—C8'—C12	107.6 (12)
C2—C3—H3	118.8	C7—C8'—H8'	107.8
C5—C4—C3	117.1 (2)	C9'—C8'—H8'	107.8
C5—C4—C7	121.8 (2)	C12—C8'—H8'	107.8
C3—C4—C7	121.1 (2)	C8'—C9'—O3	110.4 (10)
C4—C5—C6	121.4 (2)	C8'—C9'—H9'1	109.6
C4—C5—H5	119.3	O3—C9'—H9'1	109.6
C6—C5—H5	119.3	C8'—C9'—H9'2	109.6
C1—C6—C5	120.2 (2)	O3—C9'—H9'2	109.6
C1—C6—H6	119.9	H9'1—C9'—H9'2	108.1
C5—C6—H6	119.9	O3—C10—C16	116.5 (2)
C8'—C7—C4	121.6 (6)	O3—C10—C11	122.6 (2)
C8—C7—C4	113.3 (2)	C16—C10—C11	121.0 (3)
C8'—C7—H7A	85.7	C10—C11—C13	118.3 (2)
C8—C7—H7A	108.9	C10—C11—C12	120.4 (2)
C4—C7—H7A	108.9	C13—C11—C12	121.2 (2)
C8'—C7—H7B	119.9	O2—C12—C11	122.2 (2)
C8—C7—H7B	108.9	O2—C12—C8	123.4 (2)
C4—C7—H7B	108.9	C11—C12—C8	114.1 (2)
H7A—C7—H7B	107.7	O2—C12—C8'	118.2 (4)
C8'—C7—H7C	106.9	C11—C12—C8'	116.4 (4)
C8—C7—H7C	128.8	C14—C13—C11	120.6 (3)
C4—C7—H7C	106.9	C14—C13—H13	119.7
H7B—C7—H7C	85.5	C11—C13—H13	119.7
C8'—C7—H7D	107.0	C13—C14—C15	119.8 (3)
C8—C7—H7D	90.8	C13—C14—H14	120.1
C4—C7—H7D	106.9	C15—C14—H14	120.1
H7A—C7—H7D	126.9	C16—C15—C14	121.1 (3)
H7C—C7—H7D	106.7	C16—C15—H15	119.5
C9—C8—C7	116.1 (3)	C14—C15—H15	119.5
C9—C8—C12	107.2 (3)	C15—C16—C10	119.3 (3)
C7—C8—C12	113.2 (3)	C15—C16—H16	120.4
C9—C8—H8	106.6	C10—C16—H16	120.4
C7—C8—H8	106.6	C1—O1—H1	109.5
C12—C8—H8	106.6	C10—O3—C9	114.6 (2)
O3—C9—C8	113.7 (3)	C10—O3—C9'	115.2 (5)
			
O1—C1—C2—C3	178.6 (2)	C10—C11—C12—C8	9.9 (3)
C6—C1—C2—C3	0.1 (3)	C13—C11—C12—C8	−167.7 (3)
C1—C2—C3—C4	−0.7 (4)	C10—C11—C12—C8'	−15.4 (8)
C2—C3—C4—C5	0.7 (3)	C13—C11—C12—C8'	167.0 (7)
C2—C3—C4—C7	−179.8 (2)	C9—C8—C12—O2	146.6 (3)
C3—C4—C5—C6	−0.2 (3)	C7—C8—C12—O2	17.2 (4)
C7—C4—C5—C6	−179.7 (2)	C9—C8—C12—C11	−38.5 (5)
O1—C1—C6—C5	−178.2 (2)	C7—C8—C12—C11	−167.8 (2)
C2—C1—C6—C5	0.4 (3)	C9—C8—C12—C8'	62.8 (11)
C4—C5—C6—C1	−0.3 (3)	C7—C8—C12—C8'	−66.6 (12)
C5—C4—C7—C8'	−93.3 (10)	C7—C8'—C12—O2	−33.5 (13)
C3—C4—C7—C8'	87.3 (10)	C9'—C8'—C12—O2	−156.6 (10)
C5—C4—C7—C8	−117.9 (3)	C7—C8'—C12—C11	165.9 (7)
C3—C4—C7—C8	62.7 (3)	C9'—C8'—C12—C11	42.9 (16)
C8'—C7—C8—C9	−55.0 (17)	C7—C8'—C12—C8	76.2 (15)
C4—C7—C8—C9	61.2 (5)	C9'—C8'—C12—C8	−46.8 (12)
C8'—C7—C8—C12	69.6 (19)	C10—C11—C13—C14	−0.5 (4)
C4—C7—C8—C12	−174.1 (2)	C12—C11—C13—C14	177.1 (2)
C7—C8—C9—O3	−171.8 (2)	C11—C13—C14—C15	0.5 (5)
C12—C8—C9—O3	60.6 (6)	C13—C14—C15—C16	−0.2 (5)
C8—C7—C8'—C9'	48.6 (12)	C14—C15—C16—C10	−0.1 (5)
C4—C7—C8'—C9'	−27 (2)	O3—C10—C16—C15	179.7 (3)
C8—C7—C8'—C12	−73 (2)	C11—C10—C16—C15	0.0 (4)
C4—C7—C8'—C12	−148.8 (6)	C16—C10—O3—C9	−162.3 (3)
C7—C8'—C9'—O3	174.9 (10)	C11—C10—O3—C9	17.4 (4)
C12—C8'—C9'—O3	−58.1 (18)	C16—C10—O3—C9'	160.5 (7)
O3—C10—C11—C13	−179.4 (2)	C11—C10—O3—C9'	−19.8 (7)
C16—C10—C11—C13	0.3 (3)	C8—C9—O3—C10	−50.6 (5)
O3—C10—C11—C12	2.9 (3)	C8—C9—O3—C9'	48.5 (9)
C16—C10—C11—C12	−177.4 (2)	C8'—C9'—O3—C10	48.9 (17)
C10—C11—C12—O2	−175.1 (2)	C8'—C9'—O3—C9	−48.2 (12)
C13—C11—C12—O2	7.3 (4)		

### Hydrogen-bond geometry (Å, º)

Cg1 is the centroid of the C1–C6 ring.

**Table:**

*D*—H···*A*	*D*—H	H···*A*	*D*···*A*	*D*—H···*A*
O1—H1···O2^i^	0.82	1.94	2.752 (2)	173
C8—H8···O1^ii^	0.98	2.39	3.166 (4)	136
C16—H16···*Cg*1^iii^	0.93	3.14	4.022 (3)	159

Symmetry codes: (i) *x*−1/2, −*y*−1/2, *z*+1/2; (ii) *x*−1/2, −*y*−1/2, *z*−1/2; (iii) *x*, *y*−1, *z*.
